# Metabolite Analysis of Hangzhou Gongmei White Tea of Different Varieties

**DOI:** 10.3390/foods14091622

**Published:** 2025-05-04

**Authors:** Cun Ao, Xiaojun Niu, Haitao Huang, Jizhong Yu, Zhiqiang Cheng

**Affiliations:** Tea Research Institute, Hangzhou Academy of Agricultural Science, Hangzhou 310024, China; aocun123@163.com (C.A.); xiaojunwords@126.com (X.N.); hchyu@126.com (J.Y.); chengzq003@163.com (Z.C.)

**Keywords:** Hangzhou white tea, variety, differential metabolites

## Abstract

To comprehensively understand the quality characteristics and key characteristic metabolites of Hangzhou Gongmei white tea (HGW), an integrated approach involving sensory evaluation, chemical composition analysis, gas chromatography–mass spectrometry (GC-MS), and liquid chromatography–mass spectrometry (LC-MS) was employed to analyse the volatile and non-volatile metabolites of tea samples from different varieties. Compared to the Fudingdabai (FD) variety, the Jiukeng (JK) and Longjing (LJ) varieties exhibited more pronounced fruity or floral aromas and stronger taste profiles. The elevated concentrations of water extracts, tea polyphenols, and complex catechins in the tea infusion contributed to its increased astringency. A multivariate analysis revealed that linalool, geraniol, 2-ethylhexanol, hexanal, methyl salicylate, linalool oxide I, (*E*)-hex-2-en-1-al, β-myrcene, (*Z*)-hex-3-en-1-ol, phenylethanol, benzaldehyde, (*E*)-citral, nonanal, and *trans*-β-ionone were the primary differential volatile metabolites in HGW. The non-volatile metabolomic analyses showed that flavonoids were the main differential metabolites in HGW from different varieties. The abundance levels of the differential non-volatile metabolites were higher in JK and LJ compared to those in FD. This study provides theoretical support for the breeding and quality improvement of Hangzhou white tea, as well as the development of flowery and fruity flavoured white tea products.

## 1. Introduction

White tea is a distinctive Chinese tea that is primarily produced in the Fujian province [1], with additional production occurring in other provinces such as Zhejiang, Yunnan, Guizhou, Hunan, and Hubei [2,3]. The numerous health benefits of white tea, including its antioxidant, anti-cancer, and bacteriostatic effects, its ability to prevent diabetes and obesity and protect the nervous system, and other health functions [4,5], have led to it being revered as “one year tea, three years medicine, seven years treasure” [6]. In recent years, there has been a notable increase in the production of white tea, with growth rates of 11.43% and 15.41% in 2021 and 2022, respectively, making it the fastest-growing of the six major tea categories [7,8]. The processing technology of white tea is relatively straightforward, involving a sequence of steps such as withering, drying, and cleaning [9]. The categorisation of white tea is based on its variety and fresh leaf grade, with the classification system including Baihaoyinzhen, Baimudan, Gongmei, and Shoumei [10,11].

The quality of white tea is influenced by several factors, including variety [12,13,14], place of origin [11], plucking season [15,16], fresh leaf quality [17,18], processing technology [19,20,21], and storage conditions [22]. Among these factors, the influence of variety and origin on quality is particularly prominent. A comparison of Yunnan white tea and Fujian white tea reveals that the former is characterised by plump buds, a darker colour, and a less sweet and refreshing taste [9]. In addition, white tea produced from oolong tea varieties has been found to possess a floral aroma [23,24]. The variation in quality is primarily attributable to the differential composition of metabolites, both volatile and non-volatile, and their respective concentrations. Volatile metabolites are responsible for the aroma quality, while non-volatile metabolites determine the flavour quality. The main aroma compounds of white tea have been identified as oct-1-en-3-ol, linalool, geraniol, phenethyl alcohol, β-ionone, α-ionone, hexanal, γ-nonalactone, nonanal, phenylacetaldehyde, (*E,Z*)-nona-2,6-dienal, 2-amylfuran, safranal, and others [25]. Linalool and its oxides, geraniol, phenylacetaldehyde, and jasmone, were found to be positively correlated with floral aroma [20], while linalool and phenylacetaldehyde were also identified as the key aroma components for the fruity and sweet aroma of Jinggu white tea [26]. In addition, dihydro-5-pentyl-2(3H)-uranone, (*E*)-6,10-dimethyl undeca-5,9-dien-2-one, and 2-pentylfuran were identified as the key aroma components of milk-flavoured white tea [27]. Furthermore, free amino acids and peptides have been identified as key contributors to umami and sweet tea flavours [6,19]. Sucrose, galactitol, and fructose were identified as the predominant sugar compounds in white tea, contributing to its sweet flavour [2]. In addition to ester-type catechins, flavonoid glycosides such as quercetin glycoside, kaempferol glycoside, and myricetin glycoside also had a bitter taste and enhanced the bitterness of caffeine [28,29].

The comprehensive utilisation of tea resources is an important direction for the development of the tea industry. The popularity of white tea has led to the development of Hangzhou white tea, which is produced using fresh leaves in the late spring, summer, and autumn months. In order to standardise and promote the development of the Hangzhou white tea industry, the “Hangzhou White Tea” group standard of the Hangzhou Tea Science Society was published [30]. Currently, there is little research on Hangzhou white tea. The quality features and characteristic metabolites of HGW, one of the main products of Hangzhou white tea, are not yet fully understood [31]. This study was the first to combine sensory evaluation and metabolomics analysis of HGW from the key varieties cultivated in Hangzhou. The study provides a theoretical foundation for enhancing the quality and optimising the processing of Hangzhou white tea.

## 2. Materials and Methods

### 2.1. Chemical Reagents

The caffeine standard was purchased from Beijing Coast Hongmeng Standard Material Technology Co., Ltd. (Beijing, China); the catechin standard was purchased from Shanghai Yuanye Biotechnology Co., Ltd. (Shanghai, China); ethyl decanoate, gallic acid, glutamic acid, stannous chloride, hydrated indenone, and Foline-phenol reagents were obtained from China National Pharmaceutical Group Co., Ltd. (Shanghai, China); the colorimetric-grade glacial acetic acid, acetonitrile, and methanol were purchased from Thermo Fisher Scientific (China) Co., Ltd. (Shanghai, China); the 50/30 μm (DVB/CAR/PDMS) extraction fibre (Supelco) and C7-C30 n-alkanes were purchased from Merck KGaA (Darmstadt, Germany).

### 2.2. Tea Sample Preparation

On 16 April 2024, fresh tea leaves were harvested from the Hangzhou Academy of Agricultural Sciences tea garden. The harvested leaves comprised seven varieties, namely, Fudingdabai (FD), Jiukeng (JK), Longjing (LJ), Yingshuang (YS), Longjing No. 43 (LJ43), Jiaming No. 1 (JM1), and Baiye No. 1 (BY1). Leaf tenderness ranged from one bud and two leaves to one bud and three leaves. A combination of solar and indoor withering was utilised, with the fresh leaves being distributed in a thin layer on the spreading trays, with a weight of 400 g per square metre. Following a period of 40 h of static withering at ambient temperature, two trays were coalesced to form a single tray. Subsequent to 18 and 26 h of withering, the trays were exposed to sunlight for a duration of 30 min each. Following a total of 50 h of withering, the trays were reunited. Following this, the trays were dried at 100 °C for 10 min, cooled for 1 h, and then heated at 80 °C for 20 min.

### 2.3. Sensory Evaluation

In accordance with the stipulations outlined in the Chinese National Standard entitled “Method for Sensory Evaluation of Tea” (GB/T 23776) [32], the sensory evaluation was conducted by three National First Level Tea Evaluators, who represent the highest level of expertise in this field. The evaluators provided descriptions and scores, with a maximum score of 100 points. A difference of one point indicated a difference in quality, while a difference of two points indicated a significant difference in quality.

### 2.4. Chemical Composition Analysis

Concentrations of free amino acids, tea polyphenols, water extracts, caffeine, and catechins in the tea infusion were measured, and their contents in the dry tea were analysed. The quantification of free amino acids was conducted by employing the indene ketone colorimetric method, while the determination of tea polyphenols was executed through the Folin-phenol colorimetric method. The extraction of water extracts was accomplished by the tea soup steaming dry and weighing method, and the liquid chromatography method was utilised for the measurement of catechins and caffeine. The detailed methods employed are delineated in reference [33].

### 2.5. Analysis of the Volatile Metabolites

The determination of volatile metabolites is based on reference [32], with slight modifications to the volatile extraction method. Firstly, 1.60 g of crushed tea powder should be weighed and transferred into a 100 mL sample bottle; then, 80 mL of 50 °C water and 10 μL of internal standard (ethyl decanoate, 0.0863 g/L) should be added. These should then be mixed well and equilibrated in a water bath at 50 °C for 10 min. The extraction fibre is then inserted for extraction for 50 min, and, finally, the sample is injected into GC-MS (Agilent 8890GC, 5973C-MS, Santa Clara, CA, USA) for desorption at 250 °C for 5 min. The gas chromatography conditions, mass spectrometry conditions, and component identification methods were consistent with those reported in the literature.

### 2.6. Analysis of the Non-Volatile Components

Non-targeted non-volatile metabolomics analysis was commissioned from Shanghai Meiji Biomedical Technology Co., Ltd. for the purpose of testing. The sample preparation, chromatography parameters, mass spectrometry parameters, and compound identification methods have been described in reference [34]. The selection of significantly different metabolites was determined based on variable weight values (VIP) obtained from the orthogonal partial least squares discriminant analysis (OPLS-DA) model and *p*-values from Student’s *t*-test. Metabolites with VIP > 1 and *p* < 0.05 were identified as significantly different metabolites.

### 2.7. Data Statistics and Graphing

Each test was repeated three times, and the resulting data were expressed as the mean ± standard deviation. Statistical analysis and significance comparison were performed using SAS 9.3. The bar chart and radar chart were generated using Origin 2021b. The principal component analysis (PCA) and OPLS-DA multivariate analyses were analysed using SIMCA 14.0. The clustering heatmap was generated using Meiji Online Cloud Platform (https://cloud.metware.cn, accessed on 28 February 2025).

## 3. Results and Discussion

### 3.1. Sensory Evaluation Results

**Figure 1:**
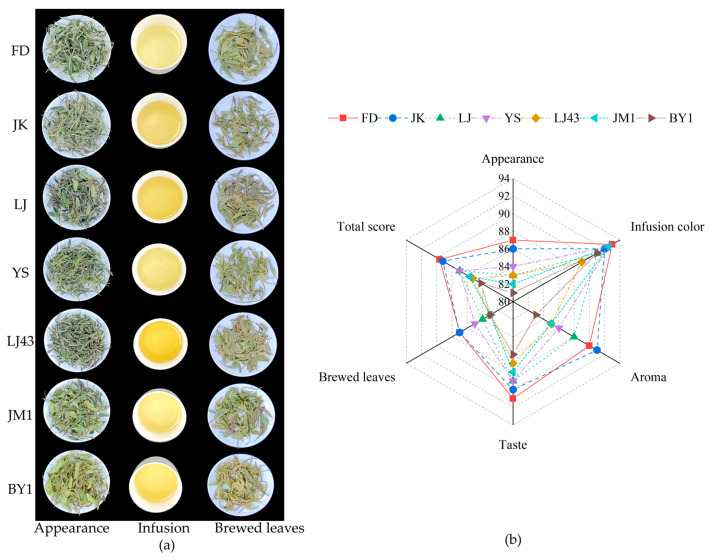
The sensory qualities of HGW. (**a**) The photos of tea samples, infusions, and brewed tea leaves. (**b**) Evaluation of each quality factor and calculation of the total score.

The tea samples, infusions and brewed tea leaves of HGW with different varieties are shown in Figure 1a. The scores for each quality factor are shown in Figure 1b, and the detailed sensory evaluation results are shown in Table S1. The best overall quality performance was exhibited by FD, the predominant variety of white tea grown in Fujian. With the exception of a marginally diminished aroma score when compared with JK, the quality factor scores were the most elevated. There was an obvious pekoe appearance, accompanied by firm buds. The aroma was characterised by a clean, pekoe-like scent with a slight floral nuance. The taste was characterised by its clean, sweet, and thin profile, which was indicative of the typical quality of Fuding white tea [11]. The low content of bitter substances such as caffeine, EGCG, ECG, and flavonoid glycosides in tea pekoe contributes to the sweetness [35]. The quality of JK was comparable, exhibiting a discernible fruity aroma and a slightly more intense flavour. The overall scores of LJ and YS were moderate, with a floral aroma in LJ. The tea sample was made with solar withering, a method that facilitates the formation of floral and fruity substances [36]. Conversely, LJ43, JM1, and BY1 exhibited low overall scores, characterised by thin buds and a green or dull aroma.

### 3.2. Analysis of Major Chemical Components

**Figure 2:**
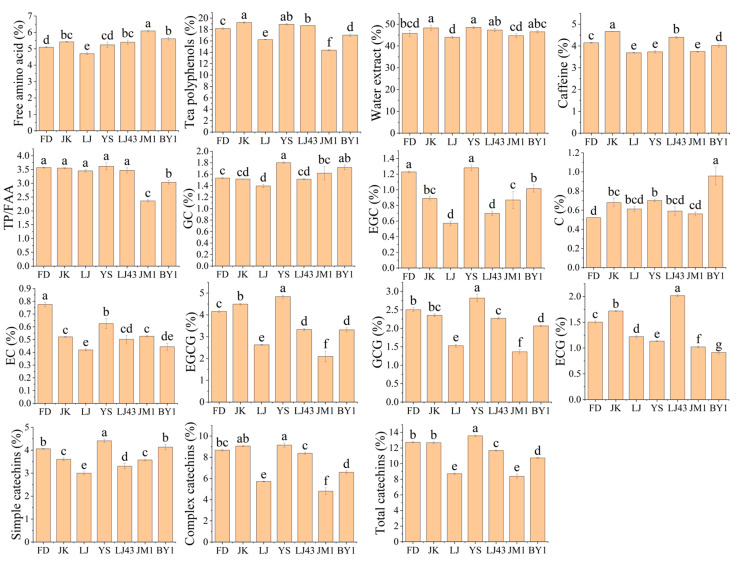
The content of main chemical compositions. Different lowercase letters indicate significant differences at *p* < 0.05. TP/FAA, the ratio of tea polyphenols to free amino acid; GC, gallocatechin; EGC, epigallocatechin; C, catechin; EC, epicatechin; EGCG, epigallocatechin gallate; GCG, gallocatechin gallate; ECG, epicatechin gallate.

The content of major chemical components in the tea samples and the concentration in the tea infusions were measured and analysed. As demonstrated in Figure 2 and Figure S1, the disparities between the varieties were substantial. The ranges of free amino acids, tea polyphenols, water extract, caffeine, and catechins in the tea samples were 4.70–6.08%, 14.37–19.24%, 43.96–48.53%, 3.69–4.66%, and 8.38–13.56%, respectively. The most significant difference was in the catechin content, which was 1.62 times higher in YS than in JM1. The content of water extract, tea polyphenols, and caffeine in JK was the highest, and the content of amino acids was moderate, rendering it rich in nutrients. Conversely, the lowest concentrations of water extract, caffeine, and free amino acids were observed in LJ. JM1 had the highest content of free amino acids, the lowest content of tea polyphenols, and the lowest ratio of polyphenols to amino acids (PAR). The content of simple catechins such as GC, EGC, and EC was lowest in LJ, and the content of complex catechins such as EGCG and GCG was also relatively low. Conversely, YS exhibited the highest concentrations of various catechins, including GC, EGC, EGCG, and GCG. The parents of YS are FD and the Yunnan large-leaf variety. Consequently, it can be hypothesised that YS has inherited the varietal characteristics of the Yunnan large-leaf variety, which has been shown to contain higher levels of polyphenolic substances [9,11]. Conversely, BY1, JM1, LJ, and LJ43 exhibited varying degrees of leaf redness and reduced catechin levels, attributable to oxidation.

The concentrations of various chemical constituents in the infusions were not entirely consistent with those found in the dry tea. LJ43 was characterised by thin buds and leaves, and the highest concentrations of water extract, free amino acids, tea polyphenols, and caffeine were observed in the tea infusion, resulting in the strongest flavour and deepest infusion colour. In contrast, the buds and leaves of JK were comparatively thick. While the dry tea contained high levels of tea polyphenols and water extracts, these concentrations were lower in the infusion. There was no astringency in the tea infusion. The FD infusion exhibited the lowest concentrations of water extract, free amino acids, and caffeine. Despite the highest PAR, the infusion colour was the lightest and the taste was sweet and thin. YS and LJ43 demonstrated significantly higher concentrations of tea polyphenols, complex catechins, and total catechins compared to the other teas. It is noteworthy that complex catechins, due to their nature, are known to elicit a bitter and astringent taste [11,23], thus contributing to the perception of a slight astringency in the tea. It is evident that the concentration of chemical constituents in tea infusions of intact buds and leaves is not only related to the content of constituents in dry tea, but is also influenced by other factors such as leaf size and thickness. The smaller the leaves, the greater the specific surface area, which is more conducive to the dissolution of internal substances. The thicker the leaves, the slower the rate of dissolution of internal substances.

### 3.3. Differential Analysis of Volatile Metabolites

**Figure 3:**
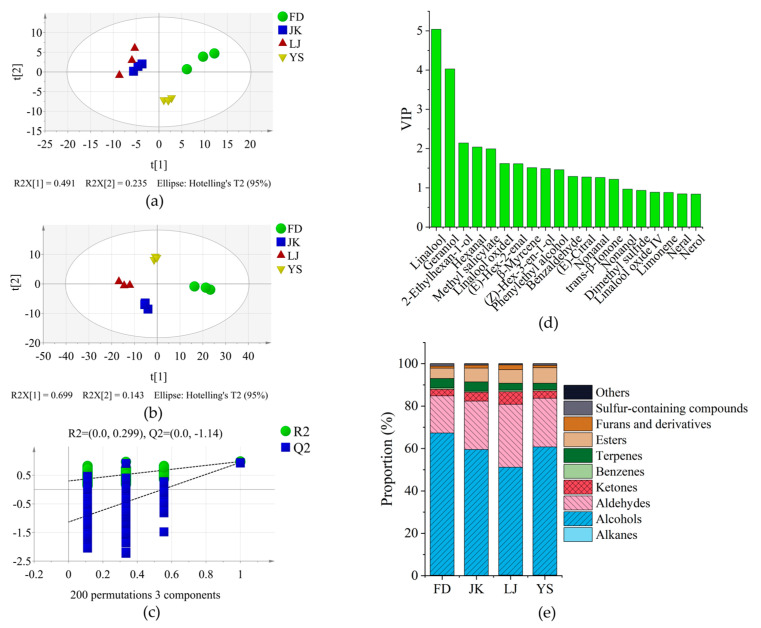
Analysis of aroma compounds in HGW. (**a**) PCA diagram. (**b**) OPLS-DA (R2Y = 0.977, Q2 = 0.937) diagram. (**c**) Cross-validation by 200-time permutation test (R2 = 0.299, Q2 = −1.14). (**d**) Aroma components of variables important in projecting. (**e**) The proportion of different types of aroma substances.

The volatile and non-volatile substances of FD, JK, LJ, and YS with high scores were measured. A total of 92 aroma components were identified through a process of matching with the NIST 20 mass spectrometry library and retention index (Table 1). The categorisation of these components was conducted based on their compositional characteristics, resulting in the identification of ten distinct categories: alcohols (27), aldehydes (23), ketones (13), esters (5), terpenes (6), alkanes (4), benzenes (5), furans (5), sulphur-containing compounds, (2) and others (2). PCA was performed on the content of different aroma components (Figure 3a). The first two principal components were found to account for 72.6% of the variability in the original data. The distance between JK and LJ was relatively close, while the other two were clearly distinguished, which was basically similar to the sensory evaluation results. The OPLS-DA method was utilised to construct a discriminant model (Figure 3b), and the four varieties were effectively discriminated, with R2Y and Q2 values of 0.977 and 0.937, respectively. The model demonstrated no signs of overfitting, as evidenced by the 200 prediction analyses (Figure 3c). The VIP analysis results (Figure 3d) demonstrated that linalool, geraniol, 2-ethylhexanol, hexanal, methyl salicylate, linalool oxide I, (*E*)-hex-2-enal, β-myrcene, (*Z*)-hex-3-en-1-ol, phenylethanol, benzaldehyde, (*E*)-citral, nonanal, and *trans*-β-ionone were the main discriminating components. It is noteworthy that the majority of these substances align with the key aroma components of Fujian white tea, as reported in the literature [25,37].

The relative content of different types aroma compounds was analysed (Figure 3e), with alcohols (50.87–67.08%), aldehydes (17.53–29.64%), and ketones (3.07–5.98%) accounting for over 85% of the total aroma content. The proportion of alcohols, terpenes, and sulphur-containing compounds in FD was the highest, while the proportion of aldehydes, ketones, and furans was the lowest. The total amount of aroma substances was also the highest, including clean aroma components such as (*Z*)-hex-3-en-1-ol, 2-ethylhexanol, (*Z*)-non-3-en-1-ol, (*E,Z*)-nona-3,6-dien-1-ol, and (*E,Z*)-nona-2,6-dienal [38]; floral aroma components such as linalool and its oxides, geraniol, and phenethyl alcohol [39]; and sweet aroma components such as dimethyl sulphide [40], α-terpineol [41], and β-myrcene [42], with higher content than other varieties. The amalgamation of these substances resulted in a clean, sweet, and slightly floral aroma. The proportion of alkanes in JK was the lowest, while the proportion of ketones, esters, and furans was relatively high. The presence of clean aroma components such as (*Z*)-hex-3-en-1-ol, hex-2-enol, hex-2-enal, and nonanal was minimal, while the content of fruit aroma components such as 2-pentylfuran [43], neral [44], octanal [45], decanal [45,46], dodecanal [46], 6-methylhept-5-en-2-one [41], and (*E*)-citral [47] was substantial. The manifestation of the aroma was fruity. The highest proportion of aldehydes, ketones, benzenes, and furans was observed in LJ, while the content of alcohols and sulphur-containing compounds was the lowest. The content of floral components such as nerolidol, jasmone, β-ionone, and geranic acid methyl ester was significantly higher than other varieties [48,49]. Furthermore, the highest concentration of hexanal, which was associated with a grassy aroma, had been detected in LJ [20,50]. Consequently, the aroma of LJ was characterised as floral and slightly green. YS was distinguished by its high ester content, accompanied by a comparatively low level of terpenes and other constituents. The proportion of most substances was moderate, and the aroma was clean and pure.

### 3.4. Differential Analysis of Non-Volatile Metabolites

**Figure 4:**
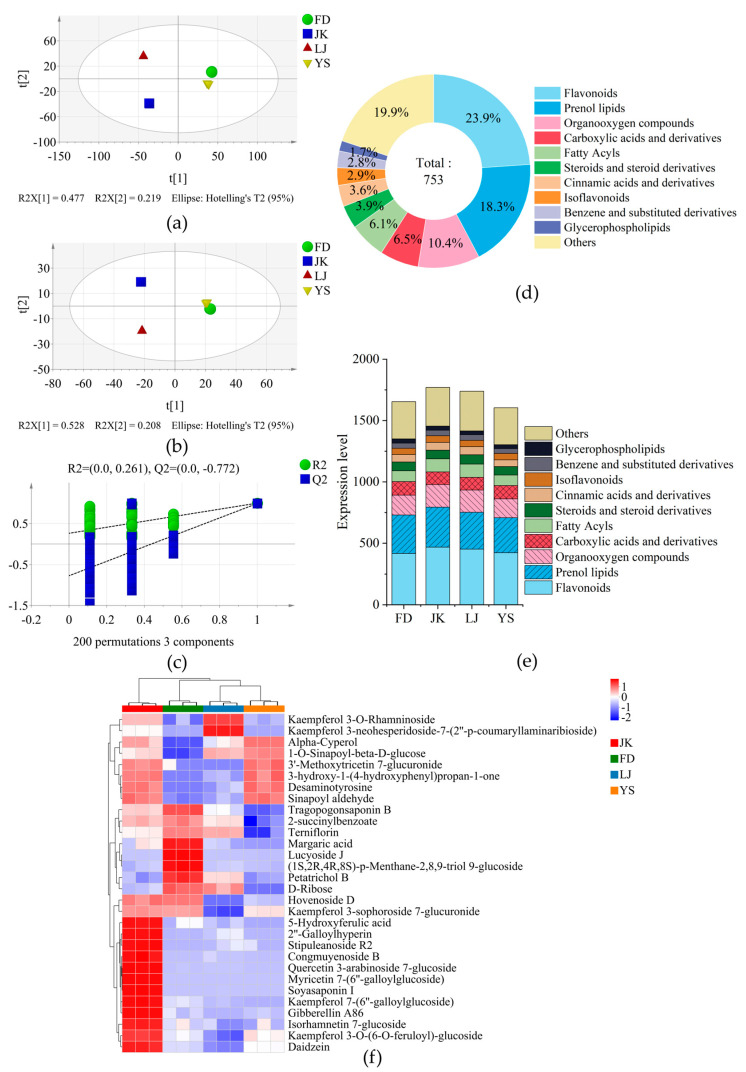
Analysis of non-volatile compounds in HGW. (**a**) PCA chart; (**b**) OPLS-DA (R2Y = 0.999, Q2 = 0.993) chart; (**c**) cross-validation by 200-time permutation test (R2 = 0.261, Q2 = −0.772); (**d**) HMDB classification of differential non-volatile metabolites; (**e**) the expression levels of different types of non-volatile metabolites; (**f**) the top 30 non-volatile metabolites with the highest VIP values.

A total of 3690 non-volatile metabolites were identified (Table S2). PCA was performed on the abundance levels of the different metabolites (Figure 4a). The distance between FD and YS was found to be relatively close, with the four varieties distributed in different quadrants. The OPLS-DA method was then utilised to construct a discriminant model (Figure 4b), which enabled the distinction of the four varieties, with R2Y and Q2 values of 0.999 and 0.993, respectively. The model was subjected to 200 prediction analyses, which revealed that it does not demonstrate overfitting (Figure 4c). The VIP > 1 and *p*-value < 0.05 criteria were used to identify 997 differential metabolites (Table S3). According to the HMDB (Human Metabolome Database) classification, only 753 metabolites were identified (Figure 4d), which were divided into 11 categories: flavonoids (180), prenol lipids (138), organooxygen compounds (78), carboxylic acids and derivatives (49), fatty acyls (46), steroids and steroid derivatives (29), cinnamic acids and derivatives (27), isoflavonoids (22), benzene and substituted derivatives (21), glycerophospholipids (13), and others (150). The differential metabolite expression levels were found to be highest in JK, while they were lowest in YS (Figure 4e).

A significant disparity in the expression levels of diverse metabolic types was identified. The expression levels of flavonoids, prenol lipids, organooxygen compounds and isoflavonoids were all highest in JK. Conversely, the highest expression levels of carboxylic acids and derivatives and glycerophospholipids were observed in FD, while the lowest expression levels of flavonoids and isoflavonoids were seen in this sample. The expression levels of fatty acyls, steroids and steroid derivatives, cinnamic acid and its derivatives, benzene and its substituted derivatives, and others were the highest in LJ, while the expression levels of carboxylic acids and their derivatives and glycerophospholipids were the lowest. The lowest expression levels of various metabolites, including prenol lipids, organic oxygen compounds, fatty acyls, steroids and steroid derivatives, cinnamic acid and its derivatives, benzene and its substituted derivatives, and others, were observed in YS.

Among the top 30 metabolites with the highest VIP values (Figure 4f), prenol lipids and flavonoid glycosides exhibited the greatest diversity, with nine and eight species, respectively. It is evident that there were substantial variations in the specific metabolites present among the various varieties. Spring white tea has been found to contain significant levels of kaempferol and myricetin [51], and their derivatives were observed to demonstrate varied responses across different varieties in this study. In JK, several flavonoids, including myricetin 7-(6″-galloylglucoside), quercetin 3-arabinoside 7-glucoside, kaempferol 3-sphoroside 7-glucuronide, kaempferol 7-(6″-galloylglucoside), and isorhamnetin 7-glucoside, were found to be higher in abundance than in other varieties. Tragopogonasaponin B, (1S, 2R, 4R, 8S)-p-menthane-2,8,9-triol-9-glucoside, and lucyoside J were prenol lipid metabolites present at a significantly higher level in FD compared to in other varieties. Kaempferol 3-neohesperidoside-7-(2″-p-coumarinamiribioside) and kaempferol 3-O-rhamninoside were significantly higher in LJ than in other varieties. There are different types of flavonoid glycosides. In this study, the eight flavonoid glycosides were different from those screened in Fujian white tea [52,53]. The content of flavonoid glycosides was higher in JK and LJ, which may increase the antioxidant activity, but may also increase the bitterness of the tea infusion [18,54], resulting in a lower sweetness compared to FD.

## 4. Conclusions

The sensory attributes, chemical compositions, and both volatile and non-volatile metabolites of HGW, derived from fresh leaves of major cultivars grown in Hangzhou during late spring, were first systematically analysed. Significant differences were observed in the sensory quality, chemical composition, and metabolite profile of white tea produced from various tea cultivars. HGW produced from the cultivars FD, JK, LJ, and YS exhibited superior quality. Specifically, FD was characterised by a clean and pekoe aroma, whereas JK and LJ predominantly exhibited fruity or floral aromas. Elevated concentrations of water extract, tea polyphenols, and complex catechins were associated with increased astringency in the tea infusion. A multivariate analysis identified linalool, geraniol, 2-ethylhexanol, hexanal, methyl salicylate, linalool oxide I, (*E*)-hex-2-enal, β-myrcene, (*Z*)-hex-3-en-1-ol, phenylethyl alcohol, benzaldehyde, (*E*)-citral, nonanal, and *trans*-β-ionone as the principal differential components of HGW. Among the cultivars, 753 differential non-volatile metabolites were divided into 11 categories, with flavonoids being the most frequent. The abundance levels of the differential non-volatile metabolites were higher in JK and LJ compared to FD. This study provided theoretical support for the breeding and quality improvement of Hangzhou white tea, as well as the development of flowery and fruity flavoured white tea products. The next step will be to develop precise processing technology for flowery and fruity flavoured white tea with JK or LJ. The flavour characteristics and degradation mechanism of these flavonoid glycosides can also be studied to improve the sweetness of white tea.

## Figures and Tables

**Table 1:** The content of volatile components in HGW of different varieties (μg/L).

Components	RI_r_	RI_c_	CAS	FD	JK	LJ	YS
**Alkanes**							
Octane	800	799	111-65-9	0.50 ± 0.07 a	0.00 ± 0.00 c	0.39 ± 0.04 b	0.37 ± 0.03 b
2,2,4,6,6-Pentamethylheptane	943	954	13475-82-6	0.34 ± 0.05 a	0.18 ± 0.02 b	0.32 ± 0.06 a	0.35 ± 0.03 a
3-Methylnonane	965	961	5911-04-6	0.43 ± 0.02 a	0.19 ± 0.02 c	0.28 ± 0.05 b	0.43 ± 0.03 a
Decane	1000	999	124-18-5	1.12 ± 0.04 a	0.56 ± 0.03 c	0.66 ± 0.09 c	0.98 ± 0.08 b
				2.40 ± 0.03 a	0.94 ± 0.06 c	1.66 ± 0.22 b	2.13 ± 0.16 a
**Alcohols**							
Pent-1-en-3-ol	1165	1170	616-25-1	1.11 ± 0.29 a	0.74 ± 0.26 ab	0.66 ± 0.08 b	0.78 ± 0.16 ab
Pentanol	1259	1256	71-41-0	1.20 ± 0.20 a	0.75 ± 0.14 b	0.90 ± 0.20 b	0.76 ± 0.01 b
(*Z*)-Pent-2-en-1-ol	1334	1326	1576-95-0	1.61 ± 0.31 a	0.90 ± 0.13 bc	0.82 ± 0.21 c	1.29 ± 0.10 ab
Hexanol	1361	1355	111-27-3	9.33 ± 1.33 a	5.02 ± 0.20 b	4.48 ± 0.66 b	5.67 ± 0.34 b
(*Z*)-Hex-3-en-1-ol	1391	1391	928-96-1	17.00 ± 2.83 a	4.89 ± 0.46 b	7.68 ± 1.27 b	7.17 ± 0.36 b
Octan-3-ol	1393	1397	589-98-0	0.33 ± 0.07 a	0.24 ± 0.04 ab	0.16 ± 0.05 b	0.15 ± 0.01 b
(*E*)-Hex-2-en-1-ol	1412	1412	928-95-0	3.22 ± 0.36 a	1.52 ± 0.09 c	1.82 ± 0.24 c	2.57 ± 0.27 b
Linalool oxideⅠ	1450	1451	34995-77-2	37.00 ± 5.58 a	17.12 ± 2.30 c	14.43 ± 2.94 c	24.38 ± 1.10 b
Oct-1-en-3-ol	1452	1456	3391-86-4	2.96 ± 0.27 ab	3.33 ± 0.30 a	3.29 ± 0.61 a	2.32 ± 0.04 b
Heptanol	1456	1461	111-70-6	6.38 ± 0.69 a	3.00 ± 0.17 c	2.89 ± 0.47 c	3.96 ± 0.21 b
6-Methylhept-5-en-2-ol	1467	1468	1569-60-4	1.90 ± 0.21 a	0.62 ± 0.06 b	0.72 ± 0.26 b	0.91 ± 0.08 b
2-Ethylhexan-1-ol	1496	1496	104-76-7	41.82 ± 4.60 a	22.73 ± 1.76 c	9.66 ± 1.18 d	34.52 ± 2.02 b
Linalool	1552	1558	78-70-6	358.29 ± 46.85 a	172.38 ± 18.51 b	118.07 ± 19.67 c	217.22 ± 9.60 b
Octanol	1562	1564	111-87-5	5.74 ± 0.59 a	3.43 ± 0.28 c	4.31 ± 0.69 bc	4.85 ± 0.23 ab
Hotrienol	1621	1617	29957-43-5	0.57 ± 0.08 a	0.52 ± 0.11 a	0.28 ± 0.05 b	0.30 ± 0.02 b
Nonanol	1664	1666	143-08-8	14.46 ± 1.24 a	7.69 ± 0.63 c	6.55 ± 0.71 c	10.82 ± 0.48 b
(*Z*)-Non-3-en-1-ol	1693	1691	10340-23-5	3.13 ± 0.24 a	1.61 ± 0.10 bc	0.87 ± 0.76 c	2.37 ± 0.19 ab
α-Terpineol	1706	1706	98-55-5	2.34 ± 0.21 a	1.52 ± 0.13 b	0.85 ± 0.16 c	0.87 ± 0.05 c
(*E,Z*)-Nona-3,6-dien-1-ol	1762	1758	56805-23-3	0.76 ± 0.06 a	0.31 ± 0.05 c	0.19 ± 0.02 d	0.51 ± 0.02 b
Decanol	1766	1765	112-30-1	0.63 ± 0.04 a	0.32 ± 0.11 b	0.45 ± 0.08 b	0.32 ± 0.03 b
Linalool oxide Ⅳ	1750	1771	39028-58-5	6.38 ± 1.24 a	1.75 ± 0.15 b	2.32 ± 0.25 b	2.34 ± 0.12 b
Nerol	1808	1807	106-25-2	8.06 ± 1.16 a	5.59 ± 0.55 b	4.25 ± 0.72 bc	3.60 ± 0.17 c
Isogeraniol	1820	1820	5944-20-7	2.17 ± 0.24 a	1.03 ± 0.10 bc	0.81 ± 0.14 c	1.17 ± 0.08 b
Geraniol	1857	1859	106-24-1	144.45 ± 20.96 a	134.57 ± 17.94 a	75.91 ± 12.96 b	71.49 ± 3.38 b
Benzyl alcohol	1898	1890	100-51-6	4.11 ± 0.57 a	3.50 ± 0.45 ab	3.16 ± 0.43 b	4.34 ± 0.20 a
Phenylethyl alcohol	1935	1926	60-12-8	25.81 ± 3.92 a	9.45 ± 1.26 c	8.87 ± 1.36 c	14.92 ± 0.57 b
Nerolidol	2050	2055	40716-66-3	2.07 ± 0.08 b	2.60 ± 0.34 a	2.87 ± 0.41 a	1.23 ± 0.03 c
				702.84 ± 92.27 a	407.12 ± 45.96 b	277.27 ± 45.54 c	420.83 ± 18.15 b
**Aldehydes**							
2-Methylbutanal	920	918	96-17-3	3.01 ± 0.48 a	1.70 ± 0.13 b	1.38 ± 0.22 b	3.25 ± 0.17 a
3-Methylbutanal	918	921	590-86-3	1.36 ± 0.28 a	0.87 ± 0.03 b	0.87 ± 0.15 b	1.46 ± 0.11 a
Pentanal	982	985	110-62-3	2.11 ± 0.37 a	1.41 ± 0.12 b	1.80 ± 0.31 ab	1.55 ± 0.09 b
Hexanal	1089	1089	66-25-1	27.13 ± 3.38 ab	23.43 ± 0.34 b	32.89 ± 6.50 a	21.18 ± 0.60 b
(*E*)-Pent-2-enal	1134	1139	1576-87-0	1.83 ± 0.42 a	1.36 ± 0.13 a	1.67 ± 0.23 a	1.53 ± 0.06 a
Heptanal	1194	1193	111-71-7	10.13 ± 0.57 a	7.94 ± 0.11 b	7.37 ± 0.90 b	9.41 ± 0.23 a
(*E*)-Hex-2-enal	1218	1230	6728-26-3	56.88 ± 5.11 a	38.61 ± 3.44 b	40.75 ± 7.02 b	46.53 ± 3.02 b
(*Z*)-Hept-4-enal	1252	1250	929-22-6	0.70 ± 0.18 b	0.41 ± 0.09 c	0.49 ± 0.03 bc	1.09 ± 0.15 a
Octanal	1296	1296	124-13-0	3.81 ± 0.20 b	4.15 ± 0.12 b	5.62 ± 1.21 a	3.75 ± 0.13 b
Nonanal	1400	1401	124-19-6	20.70 ± 0.72 a	15.27 ± 1.13 b	16.47 ± 1.65 b	22.82 ± 1.34 a
(*E,E*)-Hexa-2,4-dienal	1409	1415	142-83-6	1.75 ± 0.18 ab	1.19 ± 0.06 c	1.96 ± 0.47 a	1.31 ± 0.05 bc
(*E*)-Oct-2-enal	1441	1439	2548-87-0	2.22 ± 0.12 c	2.43 ± 0.24 bc	3.23 ± 0.49 a	2.80 ± 0.06 ab
(*E,E*)-Hepta-2,4-dienal	1507	1505	4313-03-5	4.98 ± 0.88 a	3.24 ± 0.96 a	3.87 ± 1.30 a	3.63 ± 0.27 a
Decanal	1510	1506	112-31-2	0.00 ± 0.00 b	3.06 ± 0.40 a	2.70 ± 0.31 a	0.00 ± 0.00 b
Benzaldehyde	1536	1539	100-52-7	14.51 ± 1.55 b	17.84 ± 1.29 a	12.14 ± 2.14 b	14.33 ± 0.52 b
(*E*)-Non-2-enal	1548	1547	18829-56-6	1.32 ± 0.06 a	1.30 ± 0.16 a	1.11 ± 0.23 a	1.24 ± 0.12 a
(*E,Z*)-Nona-2,6-dienal	1599	1599	557-48-2	0.78 ± 0.03 a	0.48 ± 0.05 c	0.33 ± 0.02 d	0.61 ± 0.05 b
β-Cyclocitral	1638	1634	432-25-7	6.08 ± 0.60 a	4.25 ± 0.33 b	6.22 ± 0.87 a	6.35 ± 0.28 a
(*E*)-Dec-2-enal	1655	1655	3913-81-3	0.22 ± 0.01 c	0.33 ± 0.04 b	0.44 ± 0.04 a	0.22 ± 0.01 c
Benzeneacetaldehyde	1663	1659	122-78-1	8.51 ± 1.38 a	8.05 ± 0.96 ab	6.45 ± 1.09 b	9.16 ± 0.43 a
2-Butyloct-2-enal	1659	1678	13019-16-4	0.62 ± 0.07 b	1.14 ± 0.17 a	1.24 ± 0.26 a	0.60 ± 0.02 b
Neral	1694	1694	106-26-3	3.72 ± 0.30 b	5.02 ± 0.36 a	3.78 ± 0.32 b	1.79 ± 0.09 c
(*E*)-Citral	1744	1745	141-27-5	10.71 ± 1.07 b	12.43 ± 1.00 a	8.67 ± 0.90 c	5.25 ± 0.17 d
				183.09 ± 16.29 a	155.93 ± 7.14 a	161.46 ± 25.88 a	159.87 ± 6.05 a
**Ketones**							
2-Methylpentan-3-one	1007	1003	565-69-5	0.08 ± 0.01 b	0.09 ± 0.00 ab	0.12 ± 0.04 a	0.07 ± 0.01 b
Pent-1-en-3-one	1025	1026	1629-58-9	1.39 ± 0.31 a	0.82 ± 0.16 b	0.90 ± 0.16 b	1.52 ± 0.06 a
Heptan-2-one	1189	1190	110-43-0	0.47 ± 0.08 c	0.88 ± 0.12 b	1.22 ± 0.24 a	0.37 ± 0.03 c
6-Methylheptan-2-one	1247	1245	928-68-7	0.21 ± 0.05 b	0.21 ± 0.05 b	0.34 ± 0.08 a	0.14 ± 0.03 b
Octan-3-one	1257	1260	106-68-3	0.30 ± 0.04 c	0.38 ± 0.02 b	0.45 ± 0.03 a	0.22 ± 0.01 d
2,2,6-Trimethylcyclohexanone	1327	1323	2408-37-9	1.19 ± 0.23 b	2.48 ± 0.32 a	1.55 ± 0.04 b	1.22 ± 0.13 b
6-Methylhept-5-en-2-one	1346	1346	110-93-0	2.77 ± 0.22 a	2.97 ± 0.15 a	2.90 ± 0.51 a	1.82 ± 0.09 b
Oct-3-en-2-one	1416	1416	1669-44-9	1.20 ± 0.06 b	1.80 ± 0.11 a	2.10 ± 0.33 a	1.12 ± 0.11 b
Octa-3,5-dien-2-one	1522	1531	38284-27-4	5.68 ± 0.38 a	5.23 ± 0.50 a	5.42 ± 0.79 a	4.26 ± 0.14 b
(*E,E*)-Octa-3,5-dien-2-one	1573	1583	30086-02-3	2.11 ± 0.36 a	1.78 ± 0.25 ab	1.60 ± 0.31 ab	1.46 ± 0.06 b
α-Ionone	1863	1865	127-41-3	2.77 ± 0.40 a	2.15 ± 0.12 b	2.14 ± 0.37 b	1.28 ± 0.09 c
*trans*-β-Ionone	1958	1954	79-77-6	13.72 ± 1.96 a	9.41 ± 1.18 b	13.22 ± 0.60 a	10.54 ± 0.43 b
Jasmone	1969	1959	488-10-8	0.24 ± 0.03 b	0.27 ± 0.05 b	0.40 ± 0.08 a	0.00 ± 0.00 c
				32.13 ± 3.63 a	28.47 ± 2.00 ab	32.36 ± 3.32 a	24.01 ± 1.00 b
**Benzenes**							
Toluene	1047	1045	108-88-3	1.95 ± 0.20 a	0.96 ± 0.10 c	1.26 ± 0.26 bc	1.51 ± 0.09 b
Ethylbenzene	1126	1130	100-41-4	0.48 ± 0.05 a	0.26 ± 0.04 b	0.40 ± 0.12 ab	0.44 ± 0.02 a
1,3-Dimethylbenzene	1137	1138	108-38-3	0.23 ± 0.00 b	0.14 ± 0.02 c	0.28 ± 0.02 a	0.26 ± 0.02 ab
p-Xylene	1142	1145	106-42-3	0.83 ± 0.11 a	0.65 ± 0.10 a	0.70 ± 0.22 a	0.92 ± 0.07 a
p-Cymene	1280	1276	99-87-6	3.19 ± 0.46 a	1.47 ± 0.38 b	1.23 ± 0.26 b	1.18 ± 0.05 b
				6.69 ± 0.72 a	3.49 ± 0.37 b	3.87 ± 0.82 b	4.31 ± 0.10 b
**Terpenes**							
β-Myrcene	1160	1165	123-35-3	28.51 ± 4.73 a	18.51 ± 1.62 b	11.23 ± 2.30 c	13.09 ± 0.55 c
α-Terpinene	1188	1181	99-86-5	1.27 ± 0.10 a	0.74 ± 0.02 b	0.39 ± 0.04 d	0.53 ± 0.04 c
Limonene	1203	1201	5989-27-5	10.04 ± 1.33 a	5.70 ± 0.18 b	3.54 ± 0.67 c	4.60 ± 0.24 bc
*trans*-β-Ocimene	1235	1239	3779-61-1	5.16 ± 0.92 a	3.95 ± 0.50 b	1.86 ± 0.34 c	2.33 ± 0.16 c
2,6-Dimethylocta-2,4,6-triene	1382	1382	673-84-7	1.69 ± 0.26 a	1.01 ± 0.13 b	0.77 ± 0.17 bc	0.60 ± 0.04 c
Calamenene	1849	1846	483-77-2	0.00 ± 0.00 b	0.24 ± 0.01 a	0.26 ± 0.04 a	0.00 ± 0.00 b
				46.67 ± 7.19 a	30.16 ± 2.17 b	18.06 ± 3.36 c	21.16 ± 0.86 c
**Esters**							
Hexyl acetate	1282	1278	142-92-7	0.08 ± 0.01 b	0.04 ± 0.00 c	0.14 ± 0.02 a	0.04 ± 0.01 c
Hexyl 2-methylbutyrate	1438	1433	10032-15-2	0.08 ± 0.00 b	0.36 ± 0.03 a	0.41 ± 0.08 a	0.15 ± 0.02 b
Geranic acid methyl ester	1700	1705	2349-14-6	0.79 ± 0.18 bc	1.18 ± 0.15 b	1.81 ± 0.41 a	0.51 ± 0.07 c
Methyl salicylate	1798	1793	119-36-8	49.42 ± 2.80 a	42.70 ± 4.40 b	31.98 ± 3.36 c	49.86 ± 2.69 a
*cis*-Hex-3-enyl benzoate	2148	2150	25152-85-6	0.16 ± 0.01 b	0.19 ± 0.03 b	0.31 ± 0.03 a	0.28 ± 0.03 a
				50.53 ± 2.82 a	44.47 ± 4.58 a	34.65 ± 3.82 b	50.83 ± 2.62 a
**Furans and derivatives**							
2-Methylfuran	881	873	534-22-5	0.35 ± 0.05 a	0.23 ± 0.02 b	0.27 ± 0.06 b	0.20 ± 0.01 b
2-Ethylfuran	953	955	3208-16-0	3.49 ± 0.41 ab	2.86 ± 0.27 bc	3.87 ± 0.81 a	2.17 ± 0.15 c
2-n-Butylfuran	1130	1135	4466-24-4	0.37 ± 0.04 b	0.48 ± 0.01 b	0.76 ± 0.14 a	0.35 ± 0.06 b
2-Pentylfuran	1234	1237	3777-69-3	5.15 ± 0.23 ab	5.15 ± 0.32 ab	6.60 ± 1.50 a	4.39 ± 0.25 b
*cis*-2-(Pent-2-enyl)furan	1296	1308	70424-13-4	1.00 ± 0.27 a	0.67 ± 0.11 ab	0.63 ± 0.05 b	0.88 ± 0.17 ab
				10.36 ± 0.76 ab	9.40 ± 0.40 b	12.12 ± 2.53 a	8.00 ± 0.53 b
**Sulphur-containing compounds**							
Dimethyl sulphide	757	750	75-18-3	9.92 ± 1.39 a	3.36 ± 0.25 c	2.18 ± 0.31 c	4.82 ± 0.12 b
Dimethyl Sulphoxide	1579	1583	67-68-5	1.03 ± 0.33 a	0.51 ± 0.20 b	0.26 ± 0.04 bc	0.00 ± 0.00 c
				10.95 ± 1.22 a	3.87 ± 0.44 b	2.44 ± 0.32 c	4.82 ± 0.12 b
**Others**							
3,5-bis(1,1-Dimethylethyl)phenol	2310	2326	1138-52-9	0.81 ± 0.15 a	0.47 ± 0.09 b	0.34 ± 0.06 b	0.48 ± 0.04 b
Geranic acid	2347	2356	459-80-3	0.25 ± 0.03 bc	0.56 ± 0.13 a	0.33 ± 0.05 b	0.18 ± 0.01 c
				1.05 ± 0.18 a	1.03 ± 0.18 a	0.68 ± 0.11 b	0.66 ± 0.02 b
**Total**				1046.71 ± 123.75 a	684.88 ± 59.90 b	544.56 ± 85.25 b	696.62 ± 28.10 b

RIr: retention index from NIST 20 (https://webbook.nist.gov/chemistry/, accessed on 18 April 2025); RIc: retention index calculated. Different lowercase letters indicate significant differences at *p* < 0.05.

## Data Availability

The original contributions presented in the study are included in the article/Supplementary Material, further inquiries can be directed to the corresponding author.

## Supplementary Materials

The following supporting information can be downloaded at https://www.mdpi.com/article/10.3390/foods14091622/s1: Figure S1: The concentration of main chemical compositions in tea infusion; Table S1: Sensory evaluation results of HGW from different varieties; Table S2: The expression levels of all the non-volatile metabolites; Table S3: The differential non-volatile metabolites in HGW.
