# Supplementary material for: Metabolite Analysis of Hangzhou Gongmei White Tea of Different Varieties
Source: Foods. 2025 May 4;14(9):1622. doi: 10.3390/foods14091622 (PMC12071660; doi:10.3390/foods14091622)
Supplement: Supplementary file 1 [file foods-14-01622-s001.zip › TableS1.pdf]

Table S1. Sensory evaluation results of HGW from different varieties

Variety	Appearance		Liquor color		Aroma		Taste		Brewed leaves		Total score
	Description	Score	Description	Score	Description	Score	Description	Score	Description	Score	
FD	High proportion of buds with thick pekoe, greyish green	87	Light apricot, clear and bright	93	Clean, with pekoe aroma and little flowery aroma	90	Clean, sweet and thin, with pekoe taste	91	Tender and even, yellowish green	87	89.6
JK	High proportion of buds with pekoe, greyish green and slightly dark	86	Apricot and bright	92	Clear fruity aroma	91	Mellow and barely sweet, with fruity aroma	90	Tender and even, yellowish green	87	89.2
LJ	Medium proportion of buds, greyish green with auburn	83	Light yellow and bright	91	Clean and high,with flowery aroma and a little grass odor	88	Mellow and comparatively sweet,with flowery and a little grass aroma	89	Barely tender and even, yellowish green with light red	84	87.0
YS	Low proportion of buds, green and slightly auburn	84	Light apricot, clear and bright	92.5	Clean and pure	86	Mellow and brisk,slightly astringent	89	Barely tender and even, yellowish green	85	87.0
LJ43	Medium proportion of buds, greenish auburn and slightly dark	83	Yellow and bright	89	Clean,with slight grass and dull odor	85	Mellow and thick, slightly astringent and grassy	87	Barely Tender and even, yellowish green, with red leaves	83	85.3
JM1	Low proportion of buds,green alternate with auburn	82	Light apricot, clear and bright	92.5	Pure, with a little dull odor	85	Clean and mellow, with dull odor	88	Barely tender and even, green, with red leaves	83	85.7
BY1	Low proportion of buds,yellowish green alternate with auburn	81	Light yellow and bright	91	Clean, with grass odor, less pure	83	Barely clean and mellow, with grassy odor	86	Barely tender and even, greenish yellow, with red leaves	83	84.2
