# Supplementary figures and images for: Metabolite Analysis of Hangzhou Gongmei White Tea of Different Varieties
Source: Foods. 2025 May 4;14(9):1622. doi: 10.3390/foods14091622 (PMC12071660; doi:10.3390/foods14091622)

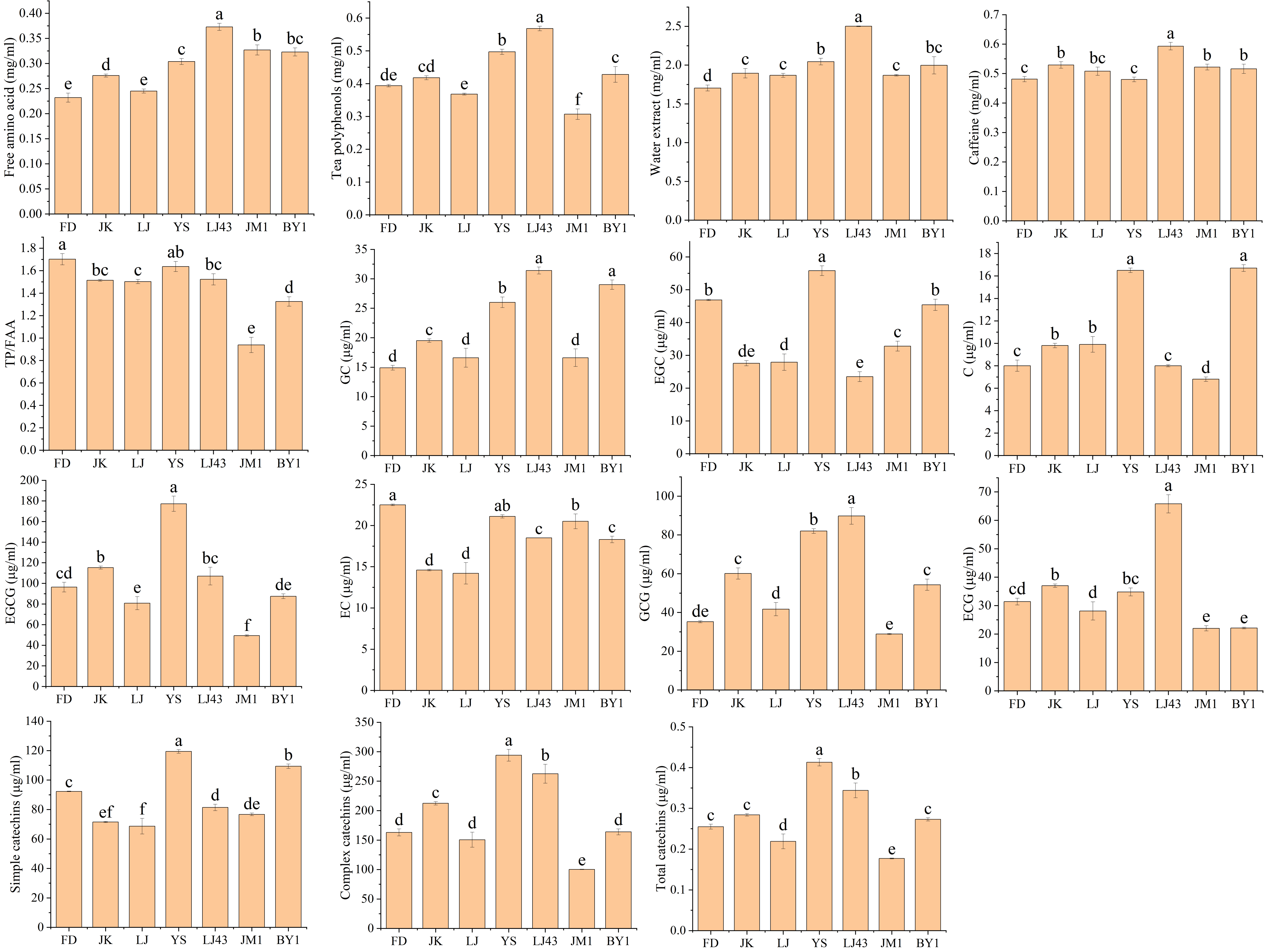

Supplement: Supplementary file 1 [file foods-14-01622-s001.zip › Figure S1.png]
